# Synthesis, Spectral and Antibacterial Studies of Binuclear Titanium(IV) / Zirconium(IV) Complexes of Piperazine Dithiosemicarbazones

**DOI:** 10.1155/S1565363303000037

**Published:** 2003

**Authors:** O. P. Pandey, S. K. Sengupta, M. K. Mishra, C. M. Tripathi

**Affiliations:** Chemistry Department D.D.U. Gorakhpur University Gorakhpur 273009 India

## Abstract

The reactions of mono(cyclopentadienyl)titanium(IV) trichloride and bis(cyclopentadienyl)titanium(IV)/ zirconium(IV) dichloride with a new class of dithiosemicarbazone, derived by condensing piperazine dithiosemicarbazide with benzaldehyde (L_1_H_2_), 2-chlorobenzaldehyde (L_2_H_2_), 4-nitrobenzaldehyde (L_3_H_2_) or salicylaldehyde (L_4_H_4_) have been studied and different types of binuclear products, *viz*. [{CpTiCl_2_}_2_L], [{Cp_2_MCl}_2_L], ((L=L_1_, L_2_ or L_3_), [{CpTiCI}_2_L_4_] and [{Cp_2_M}_2_L_4_] (M=Yi or Zr), have been isolated. Tentative structures are proposed for these complexes based upon elemental analyses, electrical conductance, magnetic moment and spectral (electronic, IR, ^1^H and ^13^C NMR) data. Attempts have been made to establish a correlation between antibacterial activity and the structures of the products.


### Synthesis, Spectral and Antibacterial Studies of Binuclear Titanium(IV) / Zirconium(IV) Complexes of Piperazine Dithiosemicarbazones

O.P. Pandey*, S.K. Sengupta, M.K. Mishra and C.M.Tripathi

*Chemistry Department, D.D.U.Gorakhpur University, Gorakhpur-273009, India*
*E-mail :* sengupta2002@yahoo.co.in

(Received: September 16, 2002; Accepted: December 2, 2002)

**ABSTRACT**

The reactions of mono(cyclopentadienyl)titanium(IV) trichloride and bis(cyclopentadienyl)titanium(IV)/ zirconium(IV) dichloride with a new class of dithiosemicarbazone, derived by condensing piperazine dithiosemicarbazide with benzaldehyde ($L_1H_2$), 2-chlorobenzaldehyde ($L_2H_2$), 4-nitrobenzaldehyde ($L_3H_2$) or salicylaldehyde ($L_4H_4$) have been studied and different types of binuclear products, *viz.* $[\{CpTiCl_2\}_2L]$, $[\{Cp_2MCl\}_2L]$, ((L=$L_1$, $L_2$ or $L_3$), $[\{CpTiCl\}_2L_4]$ and $[\{Cp_2M\}_2L_4]$ (M=Ti or Zr), have been isolated. Tentative structures are proposed for these complexes based upon elemental analyses, electrical conductance, magnetic moment and spectral (electronic, IR, $^1$H and $^{13}$C NMR) data. Attempts have been made to establish a correlation between antibacterial activity and the structures of the products.

### INTRODUCTION

Thiosemicarbazones and their metal complexes are of considerable current interest because of their potentially beneficial pharmacological properties and a wide variation in their modes of bonding and stereochemistry /1-6/. Heterocyclic thiosemicarbazones exercise their beneficial therapeutic properties in mammalian cells by inhibiting ribonucleotide reductase, a key enzyme in the synthesis of DNA precursors /7/. Their ability to provide this inhibitory action is thought to be owing to coordination *via* their N – N – S tridentate ligating system. Recent developments in the structural nature of metal complexes of heterocyclic thiosemicarbazones are correlated with their biological activities. It has been suggested that the stereochemistries and activities of complexes often depend upon the nature of N(4) substituents and on groups attached to N(1). Recently, a number of papers have also appeared /4/ on bis(thiosemicarbazone) derivatives (derived from dialdehyde or diketons) in which two thiosemicarbazone moieties are connected by their imine nitrogens, to N(1) carbon skeleton. However, very few reports are available on bis(thiosemicarbazones) in which two thiosemicarbazone moieties are connected by their amide nitrogens, N(4). West *et al.* named /8/ ligands of the second category as dithiosemicarbazones in order to differentiate it from the first category. They also reported /8/ Co(II), Ni(II) and Cu(II) complexes of such types of ligands, *viz.* N, N'-dimethylethylene diamine or piprazine dithiosemicarbazones.

The present paper describes the synthesis and characterization of titanium(IV) / zirconium(IV) derivatives with new ligands derived from piperazine dithiosemicarbazide with benzaldehyde, 2-chlorobenzaldehyde, 4-nitrobenzaldehyde or salicylaldehyde. The structure of ligands (I) is shown below.

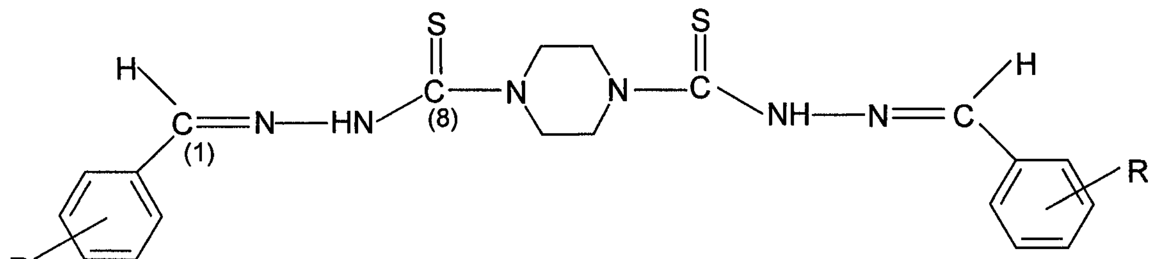

(I)

[ R = H ($L_1H_2$), 2 – Cl ($L_2H_2$), 4 – $NO_2$ ($L_3H_2$) or 2 – OH ($L_4H_2$)]

### EXPERIMENTAL

All reactions were carried out under strictly anhydrous conditions. THF was dried by heating under reflux over sodium wire. The $Et_3N$ was purified by published methods /9/. Mono(cyclopentadienyl)titanium(IV) trichloride was prepared from $Cp_2TiCl_2$ and $TiCl_4$ in p-xylene /10/. Bis(cyclopentadienyl)titanium(IV)/ zirconium(IV) dichloride were prepared /11/ by heating CpNa with appropriate metal chloride in a $N_2$ atmosphere. Elemental analyses and physical measurements were made as noted earlier /7/. Piperazine dithiosemicarbazide was prepared as reported by West *et al.* /8/.

### Preparation of dithiosemicarbazones

Piperazine dithiosemicarbazide (0.01 mol) and appropriate aldehyde (benzaldehyde, 2-chlorobenzaldehyde, 4-nitrobenzaldehyde or salicylaldehyde) (0.02 mol) were dissolved in ethanol (30 $cm^3$) and to this 2-3 drops of glacial acetic acid was added. The mixture was refluxed for 7-8 hours. The solution was concentrated to *ca.* 15 $cm^3$ and light petroleum (b.p. 60-80$^0$) (~10 $cm^3$) was added. The resulting solid was filtered, washed with cold i-PrOH and dried under vacuum.

$L_1H_1$: Yield ~ 72% ; Anal. Found : C, 58.3%; H, 5.3%; N, 20.2%; S, 15.6%;Calcd. for $C_{20}H_{22}N_6S_2$ : C, 58.5%; H, 5.4%; N, 20.5%; S, 15.6%. IR : 3150 $cm^{-1}$ (ν N-H), 1570 $cm^{-1}$ (ν C=N), 800 $cm^{-1}$ (ν C=S), $^1$HNMR : δ 12.60 (N-H), δ 8.18 (-CH=N-), δ 3.35($C_4H_8N_2$); $^{13}$C NMR : δ 165.0 (C-1), δ 150.0 (C-8), δ 62.8 ($C_4H_8N_2$), δ 3.35 ($C_4H_8N_2$)

$L_2H_2$ : Yield ~ 68%; Anal. Found : C, 50.0%; H, 4.1%; N,17.3%; S, 13.2%; Cl, 14.8%; Calcd. for $C_{20}H_{20}N_6S_2Cl_2$ : C, 50.1%; H,4.2%; N, 17.5%; S, 13.4% Cl, 14.8%. IR : 3165 $cm^{-1}$ (ν N-H), 1565 $cm^{-1}$ (ν C=N), 795 $cm^{-1}$ (ν C=S); $^1$HNMR : δ 12.95 (N-H), δ 8.20 (-CH=N-), δ 3.32 ($C_4H_8N$); $^{13}$C NMR : δ 164.5 (C-1), δ 148.2 (C-8), δ 61.5 ($C_4H_8N_2$).

$L_3H_2$: Yield ~ 70%; Anal. found : C, 47.8%; H,3.9%; N,22.2%; S, 12.6%; Calcd. for $C_{20}H_{20}N_8S_2O_4$ : C, 48.0%; H,4.0%; N, 22.4%; S, 12.8% . IR : 3160 $cm^{-1}$ (ν N-H), 1560 $cm^{-1}$ (ν C=N), 790 $cm^{-1}$ (ν C=S); $^1$HNMR : δ 12.70 (N-H), δ 8.20 (-CH=N-), δ 3.30 ($C_4H_8N$); $^{13}$C NMR : δ 164.2 (C-1), δ 149.0 (C-8), δ 61.8 ($C_4H_8N_2$)

$L_4H_4$: Yield ~ 78%; Anal. found : C, 54.1%; H,5.0%; N,18.7%; S, 14.5%; Calcd. for $C_{20}H_{22}N_6S_2O_2$ : C, 54.3%; H,5.0%; N, 19.0%; S, 14.5% .IR : 3150 $cm^{-1}$ (νN-H), 1565 $cm^{-1}$ (νC=N), 805 $cm^{-1}$ (νC=S); $^1$HNMR; δ 12.85 (N-H), δ 8.21 (-CH=N-), δ 3.32 ($C_4H_8N$); $^{13}$C NMR : δ 164.8 (C-1), δ 149.2 (C-8), δ 62.0 ($C_4H_8N_2$).

### Reactions of $Cp_2MCl_2$ and $CpTiCl_3$ with dithiosemicarbazones

To a solution of bis(cyclopentadienyl)titanium(IV)/zirconium(IV) dichloride or mono(cyclopentadienyl)titanium(IV) trichloride (20 mmol) in dry THF (*ca.* 40 $cm^3$) was added the appropriate dithiosemicarbazone (10 mmol). To the resulting clear solution, $Et_3N$ (20 mmol) was added and the mixture was stirred for *ca.* 12-22 h at room temperature. Precipitated $Et_3NHCl$ was removed by filtration and the volume of the solution was reduced to *ca.* 15 $cm^3$ under reduced pressure. Light petroleum (b.p. 60-80 $^0$C) (10 $cm^3$) was then added. The colored precipitate thus obtained was thoroughly washed with $Et_2O$ and recrystallized from 1:1 THF: $Et_2O$.

The details and important physical characteristics of new compounds are recorded in Table 1. The nuclear magnetic resonance spectra of ligands and the complexes were recorded on a Bruker 400 spectrometer. Chemical shifts are expressed relative to an internal reference TMS (1% by volume).

### RESULTS AND DISCUSSION

A systematic study of the reactions of bis(cyclopentadienyl)titanium(IV)/ zirconium(IV) dichloride and mono(cyclopentadienyl)titanium(IV) trichloride with piperazine dithiosemicarbazones (molar ratio 2:1, respectively) in anhydrous THF in the presence of triethylamine may be represented by the following equations:

$$2\,Cp_2MCl_2 + LH_2 \xrightarrow{THF} [\{Cp_2MCl\}_2(L)]$$

$$2\,CpTiCl_3\,(LH)_2 \xrightarrow{THF} [\{CpTiCl_2\}_2(L)]$$

$$[LH_2 = L_1H_2,\ L_2H_2,\ L_3H_2]$$

**Table 1.**

Reactions of mono(cyclopentadienyl)titanium(IV) trichloride and bis(cyclopentadienyl)titanium(IV)/ zirconium(IV) dichloride with piperazine dithiosemicarbazones

| Reactants | Molar ratio | Reflux time(h) | Product/ Colour | Yield (%) | Found (calcd.) % C | H | N | S | M | Cl |
|---|---|---|---|---|---|---|---|---|---|---|
| $CpTiCl_3+L_1H_2+Et_3N$ | 2:1:2 | 15 | $[\{CpTiCl_2\}_2(L_1)]$ yellowish brown | 62 | 46.2 (46.4) | 3.7 (3.9) | 10.7 (10.8) | 8.1 (8.2) | 12.2 (12.3) | 18.2 (18.3) |
| $CpTiCl_3+L_2H_2+Et_3N$ | 2:1:2 | 18 | $[\{CpTiCl_2\}_2(L_2)]$ yellowish brown | 60 | 42.3 (42.5) | 3.1 (3.3) | 9.7 (9.9) | 7.5 (7.6) | 11.2 (11.3) | 25.0 (25.1) |
| $CpTiCl_3+L_3H_2+Et_3N$ | 2:1:2 | 15 | $[\{CpTiCl_2\}_2(L_3)]$ yellow | 58 | 41.2 (41.5) | 3.1 (3.2) | 12.7 (12.9) | 7.4 (7.4) | 11.0 (11.0) | 16.2 (16.3) |
| $CpTiCl_3+L_4H_4+Et_3N$ | 2:1:4 | 12 | $[\{CpTiCl\}_2(L_4)]$ brown | 68 | 48.6 (48.9) | 3.7 (3.8) | 11.2 (11.4) | 8.6 (8.7) | 13.0 (13.0) | 9.5 (9.6) |
| $Cp_2TiCl_2+L_1H_2+Et_3N$ | 2:1:2 | 15 | $[\{Cp_2TiCl\}_2(L_1)]$ brown | 65 | 57.3 (57.5) | 4.7 (4.8) | 9.8 (10.0) | 7.5 (7.6) | 11.4 (11.5) | 8.5 (8.5) |
| $Cp_2TiCl_2+L_2H_2+Et_3N$ | 2:1:2 | 18 | $[\{Cp_2TiCl\}_2(L_2)]$ yellowish brown | 62 | 53.0 (53.1) | 4.1 (4.2) | 9.2 (9.3) | 7.0 (7.1) | 10.5 (10.6) | 15.5 (15.7) |
| $Cp_2TiCl_2+L_3H_2+Et_3N$ | 2:1:2 | 16 | $[\{Cp_2TiCl\}_2(L_3)]$ reddish brown | 64 | 51.8 (51.9) | 4.0 (4.1) | 12.0 (12.1) | 6.6 (6.9) | 10.2 (10.3) | 7.5 (7.7) |
| $Cp_2TiCl_2+L_4H_4+Et_3N$ | 2:1:4 | 12 | $[\{Cp_2Ti\}_2(L_4)]$ brown | 70 | 60.2 (60.4) | 4.7 (4.8) | 10.5 (10.6) | 8.0 (8.1) | 12.0 (12.0) | — |
| $Cp_2ZrCl_2+L_1H_2+Et_3N$ | 2:1:2 | 18 | $[\{Cp_2ZrCl\}_2(L_1)]$ brown | 62 | 52.0 (52.1) | 4.2 (4.4) | 9.0 (9.1) | 6.7 (6.9) | 19.7 (19.8) | 7.6 (7.7) |
| $Cp_2ZrCl_2+L_2H_2+Et_3N$ | 2:1:2 | 22 | $[\{Cp_2ZrCl\}_2(L_2)]$ light brown | 58 | 48.3 (48.5) | 3.7 (3.9) | 8.4 (8.5) | 6.3 (6.5) | 18.3 (18.4) | 14.3 (14.3) |
| $Cp_2ZrCl_2+L_3H_2+Et_3N$ | 2:1:2 | 22 | $[\{Cp_2ZrCl\}_2(L_3)]$ yellowish brown | 60 | 47.2 (47.5) | 3.7 (3.8) | 11.0 (11.0) | 6.1 (6.3) | 17.9 (18.0) | 6.9 (7.0) |
| $Cp_2ZrCl_2+L_4H_4+Et_3N$ | 2:1:4 | 16 | $[\{Cp_2Zr\}_2(L_4)]$ brown | 68 | 54.3 (54.5) | 4.2 (4.3) | 9.3 (9.5) | 7.2 (7.3) | 20.6 (20.7) | — |

$L_1H_1$= Dithiosemicarbazone derived from piperazine dithiosemicarbazide and benzaldehyde

$L_2H_2$= Dithiosemicarbazone derived from piperazine dithiosemicarbazide and 2-chlorobenzaldehyde

$L_3H_3$= Dithiosemicarbazone derived from piperazine dithiosemicarbazide and 4-nitrobenzaldehyde

$L_4H_4$= Dithiosemicarbazone derived from piperazine dithiosemicarbazide and salicylaldehyde

$$2\ Cp_2MCl_2 + L_4H_4 \xrightarrow{THF} [\{Cp_2M\}_2(L_4)]$$

$$2\ CpTiCl_3 + L_4H_4 \xrightarrow{THF} [\{CpTiCl\}_2(L_4)]$$

In formulas for the complexes, L is the symbol for the dianion when both of the thiosemicarbazone moieties have lost their amide proton N(2)H in the piperazine dithiosemicarbazone ligands derived from benzaldehyde, 2-chlorobenzaldehyde and 4-nitrobenzaldehyde. $L_4$ represents the loss of both amide protons and phenolic protons from the salicylaldehyde piparazine dithiosemicarbazone .

Colours, elemental analyses and molar conductivities are listed in Table 1. Complexes are soluble in dimethylformamide, dimethylsulphoxide, nitrobenzene, tetrahydrofuran. The molar conductances of the complexes in DMF are in the range 6.0-10.0 $ohm^{-1}\ cm^2\ mol^{-1}$, which are well below the range observed for uni-univalent electrolytes in this solvent. Magnetic susceptibility measurements show that they are diamagnetic. The electronic spectra of the complexes show a broad band in the 22200-23800 $cm^{-1}$, region which can be assigned /12/ to charge–transfer, a result in accord with their $(n-1)d^0ns^0$ electronic configuration. The ligands show bands at *ca.* 35000 $cm^{-1}$ and 28000 $cm^{-1}$, which are assigned to $n \rightarrow \pi^*$ transitions associated with imine and thioamide functions of the thiosemicarbazone moieties, respectively. In their respective complexes, these bands shift slightly to higher frequencies.

### I.R.Spectra

Absorption bands occurring at *ca.* 3000 $cm^{-1}$ for ν (C-H), *ca.* 1420 $cm^{-1}$ for ν (C-C) and *ca.* 1020 $cm^{-1}$ for ν (C-H) in all the complexes are assigned to the cyclopentadienyl group and indicate that these groups are π - bonded to the metal /13/.

The i.r. spectral bands most useful for determining the coordination mode for the dithiosemicarbazones are listed in Table 2. These ligands can exist either as a thione or the thiol tautomeric forms or as an equilibrium mixture of both forms, since they have a thioamide, -NH-C(=S) function. The i.r. spectra in the solid state do not show any ν(S-H) band but exhibit a medium ν(N-H) band at *ca.* 3150 $cm^{-1}$, indicating that in the solid state, they remain mainly in the thione form. However in solution they readily convert to the thiol tautomeric form with the concomitant formation of titanium(IV) / zirconium(IV) complexes of the protonoted mercapto form of the ligands. This is indicated by the absence of –NH band in the complexes. The i.r. spectra of the complexes also show a new band at *ca.* 600-620 $cm^{-1}$ owing to conversion /4/ of C=S to $C-S^-$. The new band in the complexes at *ca.* 380-400 $cm^{-1}$ is assigned /13/ to ν(M-S) and shows that sulfur is bonded to titanium/zirconium atom. The ν(C=N) shift of the dithiosemicarbazones from 1570-1560 $cm^{-1}$ to lower energy in the spectra of complexes indicates coordination of the imine nitrogens N(1) . However, the loss of N(2)H's from the two dithiosemicarbazone moieties, by thione – thiol tautomerism produces additional carbon-nitrogen double bonds, N(2)=C(S) which is indicated by the appearance of a band at *ca.* 1590 $cm^{-1}$ in the complexes. Bands in the 460-475 $cm^{-1}$ region are assigned /13/ to ν(M-N) and support coordination of the imine nitrogens. The ν(M-Cl) bands have been assigned in the 330-315 $cm^{-1}$ region.

**Table 2**

IR spectral data of titanium(iv)/zirconium(iv) derivatives with piparazine dithiosemicarbazones

| Compound | υ(C=N) | υ(C – S⁻) | υ(M – N) | υ(M – S) | $C_5H_5$ |
|---|---|---|---|---|---|
| $[\{CpTiCl_2\}_2(L_1)]$ | 1580s, 1550m | 620m | 475m | 400m | 3000s, 1425m, 1015m |
| $[\{CpTiCl_2\}_2(L_2)]$ | 1590s, 1545m | 615m | 470m | 395m | 3010s, 1420m, 1020m |
| $[\{CpTiCl_2\}_2(L_3)]$ | 1585s, 1545m | 625m | 472m | 390m | 3000m, 1420m, 1025m |
| $[\{CpTiCl\}_2(L_4)]$ | 1590s, 1545m | 610m | 470m | 400m | 2990s, 1415m, 1020m |
| $[\{Cp_2TiCl\}_2(L_1)]$ | 1585s, 1550m | 615m | 468m | 390m | 3000m, 1425m, 1020m |
| $[\{Cp_2TiCl\}_2(L_2)]$ | 1580s, 1545m | 618m | 465m | 395m | 3010s, 1420m, 1010w |
| $[\{Cp_2TiCl\}_2(L_3)]$ | 1595s, 1540m | 620m | 475m | 385m | 3000m, 1415m, 1015m |
| $[\{Cp_2Ti\}_2(L_4)]$ | 1590s, 1550m | 625m | 470m | 395m | 3010s, 1425m, 1020m |
| $[\{Cp_2ZrCl\}_2(L_1)]$ | 1590s, 1545m | 620m | 460m | 380m | 3010s, 1420m, 1020m |
| $[\{Cp_2ZrCl\}_2(L_2)]$ | 1585s, 1540m | 615m | 460m | 385m | 2990m, 1425w, 1025m |
| $[\{Cp_2ZrCl\}_2(L_3)]$ | 1595s, 1540m | 620m | 465m | 380m | 3000m, 1420m, 1020m |
| $[\{Cp_2Zr\}_2(L_4)]$ | 1590s, 1540m | 610m | 468m | 385m | 3005m, 1410m, 1015w |

### $^{1}$H N.M.R. spectra

The $^{1}$H n.m.r. spectra of the complexes (Table 3) have been recorded in deuterated dimethylsulphoxide. The line intensities were determined by planimetric integration. A comparison of the ligands with the complexes leads to the following conclusions:

(a) The δ 6.65-6.80 signal may be assigned to the cyclopentadienyl ring protons and indicate the rapid rotation of the ring about the metal-ring axis.

(b) The signal of N(2)H is seen at *ca.* δ 12.60-12.95 in the ligands. In the complexes neither this signal nor a thiol SH signal is visible.

(c) The chemical shifts at *ca.* δ 3.35 ppm can be due to piperazine ring protons which shifted slightly downfield in the complexes.

### $^{13}$C N.M.R. spectra

The $^{13}$C n.m.r spectra of ligands and the corresponding complexes were recorded in DMSO (Table 3). The $^{13}$C resonance signals are assigned according to chemical shift theory. The compounds show cyclopentadienyl peak at *ca.* δ 117 (relative to TMS). A considerable shift takes place in the position of –C-S (*ca.* 165 ppm, ligands) and C = N (*ca.* 150 ppm, ligands) indicating coordination through the azomethine nitrogen and the thiol group. The signal for piperazine group appears at *ca.* δ 62.8 in the ligands, which shift, slightly downfield in the complexes.

**Table 3**

NMR data (δ , ppm) of titanium(IV) / zirconium(IV) derivatives with piperazine dithiosemicarbazones

| Compound | $^{1}H$ - | | | $^{13}C$ – | | | |
|---|---|---|---|---|---|---|---|
| | $\eta^5 - C_5H_5$ | $C_4H_4N_2$ | Phenyl group | -CH= N | $\eta^5 - C_5H_5$ | C(8) | C(1) |
| $[\{CpTiCl_2\}_2(L_1)]$ | 6.80s | 3.45s | 7.30s | 8.20s | 117.8 | 155.65 | 168.20 |
| $[\{CpTiCl_2\}_2(L_2)]$ | 6.75s | 3.48s | 7.60-7.75m | 8.25s | 117.5 | 158.20 | 167.20 |
| $[\{CpTiCl_2\}_2(L_3)]$ | 6.70s | 3.46s | 7.70-7.82m | 8.22s | 117.0 | 156.32 | 167.60 |
| $[\{CpTiCl\}_2(L_4)]$ | 6.78s | 3.45s | 7.72-7.85m | 8.26s | 117.2 | 155.20 | 168.15 |
| $[\{Cp_2TiCl\}_2(L_1)]$ | 6.72s | 3.50s | 7.40s | 8.18s | 117.8 | 157.50 | 167.85 |
| $[\{Cp_2TiCl\}_2(L_2)]$ | 6.74s | 3.46s | 7.48-7.65m | 8.25s | 117.4 | 158.10 | 168.00 |
| $[\{Cp_2TiCl\}_2(L_3)]$ | 6.78s | 3.48s | 7.45-7.70m | 8.20s | 117.5 | 128.50 | 168.25 |
| $[\{Cp_2Ti\}_2(L_4)]$ | 6.80s | 3.42s | 7.50-7.72m | 8.28s | 117.2 | 155.70 | 168.10 |
| $[\{Cp_2ZrCl\}_2(L_1)]$ | 6.65s | 3.45s | 7.32m | 8.16s | 116.2 | 155.25 | 168.12 |
| $[\{Cp_2ZrCl\}_2(L_2)]$ | 6.68s | 3.42s | 7.42-7.65m | 8.20s | 116.5 | 156.80 | 168.25 |
| $[\{Cp_2ZrCl\}_2(L_3)]$ | 6.65s | 3.45s | 7.40-7.70m | 8.18s | 116.8 | 156.70 | 168.10 |
| $[\{Cp_2Zr\}_2(L_4)]$ | 6.70s | 3.40s | 7.48-7.75m | 8.22s | 116.0 | 157.35 | 168.0 |

**Table 4**

Antibacterial activity of piperazine dithiosemicarbazones and their corresponding titanium(IV) / zirconium(IV) complexes

| Compound | Diameter of inhibition zone (mm) | |
|---|---|---|
| | *B. subtilis* | *E. Coli* |
| $L_1H_2$ | 8 | 9 |
| $[\{CpTiCl_2\}_2(L_1)]$ | 20 | 18 |
| $[\{CpTiCl\}_2(L_1)]$ | 15 | 12 |
| $[\{Cp_2ZrCl\}_2(L_1)]$ | 12 | 10 |
| $L_2H_2$ | 12 | 12 |
| $[\{CpTiCl_2\}_2(L_2)]$ | 22 | 20 |
| $[\{Cp_2TiCl\}_2(L_2)]$ | 18 | 16 |
| $[\{Cp_2ZrCl\}_2(L_2)]$ | 14 | 15 |
| $L_3H_2$ | 10 | 12 |
| $[\{CpTiCl_2\}_2(L_3)]$ | 20 | 18 |
| $[\{Cp_2TiCl\}_2(L_3)]$ | 16 | 14 |
| $[\{Cp_2ZrCl\}_2(L_3)]$ | 14 | 14 |
| $L_4H_4$ | 12 | 11 |
| $[\{CpTiCl\}_2(L_4)]$ | 20 | 19 |
| $[\{Cp_2Ti\}_2(L_4)]$ | 16 | 16 |
| $[\{Cp_2Zr\}_2(L_4)]$ | 13 | 12 |
| Ampicillin | 35 | 35 |

### Antibacterial Activity

The antibacterial activity of the complexes together with the parent ligands has been screened /14/ against Gram-positive *Bacillus subtilis* and Gram-negative *Escherichia coli* by the paper disk plate method at 1000 ppm conc. The inhibition zone (mm) around each disk was measured after 24 h and the results of these studies are listed in Table 4. The results lead to the following conclusions:

(a) The complexes are slightly more toxic than the parent ligands.

(b) The titanium complexes show better activity than zirconium complexes.

(c) Mono(cyclopentadienyl)titanium(IV) derivatives possess better activity than bis(cyclopentadienyl)titanium(IV) derivatives .

(d) The best activity was recorded with mono(cyclopentadienyl)titanium(IV) derivatives with ligand $L_2H_2$ *i.e.* containing –Cl group at phenyl ring of the thiosemicarbazone moieties.

On the basis of elemental analysis, electrical conductance and spectral data, the following structures for the complexes are proposed.

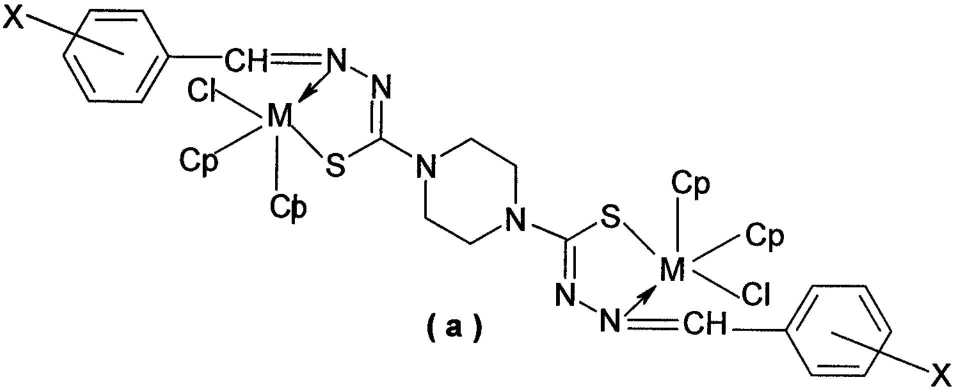

(a)

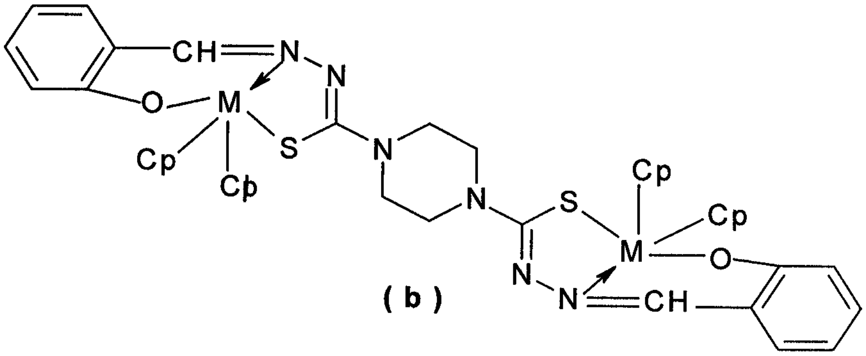

(b)

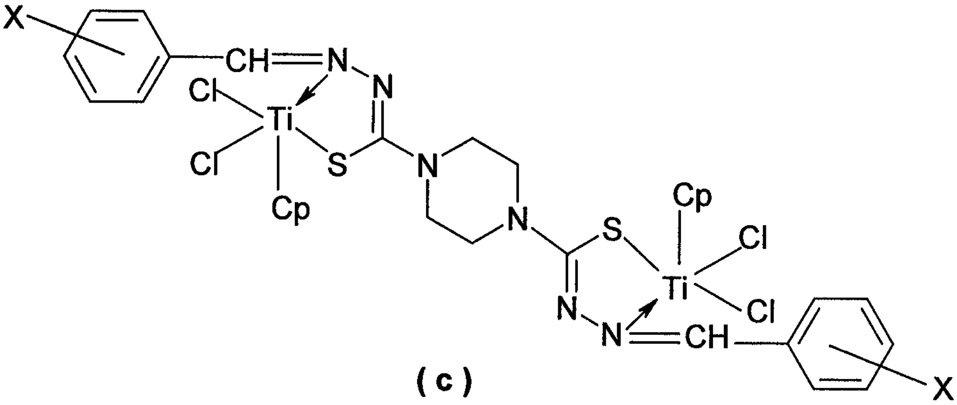

(c)

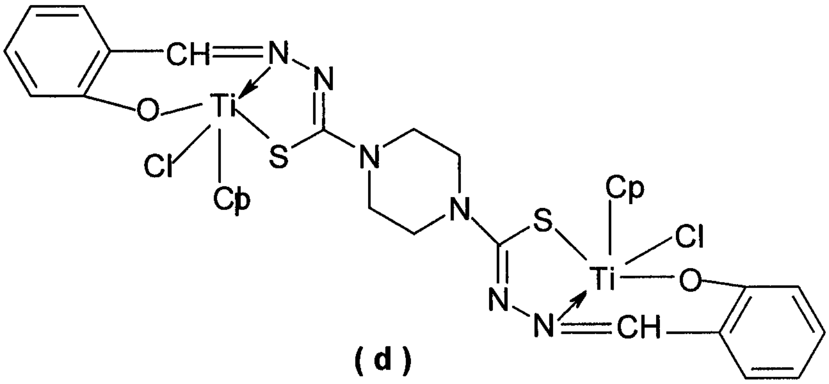

(d)

### ACKNOWLEDGEMENT

SKS thanks Prof. E. Hey-Hawkins, Universitat Leipzig, Germany for her help in recording NMR spectra during his visit to Germany under the INSA-DFG exchange program. MKM thanks the Council of Scientific & Industrial Research, New Delhi for the award of JRF.

### REFERENCES

1. D.X. West, A.E. Liberta, S.B. Padhye, R.C. Chikate, P.B. Sonawane, A.S. Kumbhar and R.G. Yerande, *Coord. Chem. Rev.*, **123**, 49 (1993).
2. D.X. West, S.B. Padhye and P.B. Sonawane, *Struct. Bond.*, **76**, 1(1991).
3. M. Akbar Ali, A.H. Mirza, A.M.S. Hossain and M. Nazimuddin, *Polyhedron,* **20** 1045 (2001) and references therein.
4. A. Castineiras, R. Carballo and T. Perez, *Polyhedron,* **20**, 441(2001) and references therein.
5. C-Y. Duan, B-M Wu and T.C.W. .Mak, *J. Chem. Soc., Dalton Trans.*, 3485 (1996).
6. J.S. Casas and M.S. Garcia-Tasende, J. Sordo, *Coord.Chem. Rev.*, **209**, 197(2000).
7. S.K. Sengupta, O.P. Pandey, B.K. Srivastava and V.K.Sharma, *Transition Met. Chem.*, **23**, 349 (1998) and reference therein.
8. J.K. Sweatingen and D.X. West, *Transition Met. Chem*, **25**, 241(2000).
9. A.I. Vogel, *A Textbook of Practical Organic Chemistry*, Longmans Green, London, 1948.
10. R.D. Gorsich, *J. Am. Chem. Soc.*, **82**, 4211(1960).
11. G. Wilkinson and J.M. Birmingham, *J. Am. Chem. Soc.*, **76**, 4281(1954).
12. R. Rai, K.D. Mishra, O.P. Pandey and S.K Sengupta, *Polyhedron*, **11**,123(1992).
13. A. Mala, A.K. Srivastava, O.P. Pandey and S.K. Sengupta, *Transition Met. Chem.*, **25**, 613(2000).
14. M. Carcelli, P. Mazza, C. Pelizzi, G. Pelizzi and F. Zani, *J. Inorg. Biochem.*, **57**, 43(1995).

